# Association of serum Klotho levels with cancer and cancer mortality: Evidence from National Health and Nutrition Examination Survey

**DOI:** 10.1002/cam4.5027

**Published:** 2022-07-16

**Authors:** Yating Qiao, Fubin Liu, Yu Peng, Peng Wang, Bing Ma, Limin Li, Changyu Si, Xixuan Wang, Ming Zhang, Fangfang Song

**Affiliations:** ^1^ Department of Epidemiology and Biostatistics, Key Laboratory of Molecular Cancer Epidemiology, Tianjin, National Clinical Research Center for Cancer, Tianjin's Clinical Research Center for Cancer Tianjin Medical University Cancer Institute and Hospital Tianjin China; ^2^ Shenzhen Prevention and Treatment Center for Occupational Diseases Shenzhen China

**Keywords:** cancer, cancer mortality, estradiol, NHANES, Serum Klotho, testosterone

## Abstract

**Background:**

Klotho has both anticancer and hormone‐like functions. But the research on Klotho and cancer is mainly based on animal experiments and small‐scale clinical research, thus we explored the association between serum Klotho and cancer and cancer mortality based on the National Health and Nutrition Survey (NHANES).

**Methods:**

Participants were employed from the NHANES 2007–2016, excluding pregnant, chronic renal insufficiency, and incomplete data of cancer questionnaire and serum Klotho level. The association of serum Klotho with cancer and mortality was analyzed by weighted Logistic regression, weighted Cox regression and competitive risk model, respectively. Correlations between serum Klotho and testosterone and estradiol levels were analyzed by Spearman correlation and restricted cubic spline respectively.

**Results:**

We found Klotho had an inverse effect with risk of pan‐cancer (all *p* < 0.02), with each unit increase in Klotho (1ug/g creatinine) associated with a 0.9%–2.2% reduction in the risk of cancer, and higher levels showing a stronger negative association (all *p*‐trend <= 0.0005). Whereas, we did not observe any association between serum Klotho level with all‐cause mortality and cancer‐specific mortality (all *p* > 0.05). Then, stratified analysis found that people aged 60–79, female, overweight and non‐Hispanic whites or Mexican Americans were less likely to develop cancer. In addition, there was a strong nonlinear and linear positive correlation of Klotho with estradiol (*p*‐nonlinear = 0.0178) and testosterone only among male participants (β = −0.513, *p* = 0.0137), respectively.

**Conclusions:**

We found an inverse association between serum Klotho and cancer, but without cancer mortality. And this effect may be partially mediated by estradiol and testosterone. Further prospective studies are needed to prove these findings.

## Graphical abstract

Serum Klotho as a new biomarker for cancer.

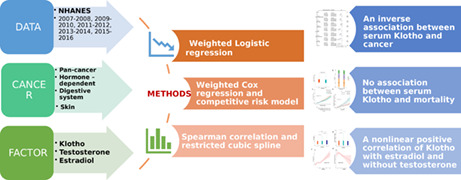

**Abbreviations**
BMI: body mass index
CI: confidence interval
IQR: Interquartile range.
NHANES: National Health and Nutrition Examination Survey
OR: odds ratio
PIR: poverty income ratio
SD: standard deviation

Lay Summary

Current researches on Klotho and cancer have focused on animal studies and small‐scale clinical research. This study was based on the US National Health and Nutrition Examination Survey of a nationally representative sample. We found Klotho had an inverse association on pan‐cancer with higher levels showing a stronger negative effect, but no association with cancer‐specific mortality. Further, we found a positive correlation between Klotho with both estradiol and testosterone (only in male participants). The causal relationship between them needs further exploration.

## 1 INTRODUCTION

Despite great advances in prevention, diagnosis and treatment, cancer remains the second leading cause of death in the United States (US)^1^, ^2^ and a public problem worldwide. There is an urgent need for better predictive programs, more accurate diagnostic criteria, and more reasonable and effective treatment programs.

In 1997 M Kuro‐o et al. pointed out that Klotho gene had a certain correlation with body aging in a research report on spontaneous hypertension.^3^ The research suggested that once mice lacked the Klotho gene, there would be functional disorders similar to human atherosclerosis, cancer and osteoporosis.^4^ Subsequent human studies also found similar dysfunction with Klotho deficiency, particularly in cancer, cardiovascular and kidney.^5^, ^6^, ^7^, ^8^ The Klotho gene mainly exists in kidney and brain tissues and produces membrane‐bound receptors and humoral regulatory factors.^9^ At present, three members of the Klotho family (α, β, γ) have been found, showing sequence similarity with members of the glycosidase family.^10^ Alpha‐Klotho, the first member discovered by accident, is different from the other two members of the Klotho family because it has a dual function as a transmembrane protein of FGF23 co‐receptor.^11^ Meanwhile, alpha‐Klotho is cleaved by secretases ADAM 10 and 17,^12^ and secreted from cells into the blood, cerebrospinal fluid and urine,^13^, ^14^, ^15^ as has not been reported for β‐Klotho or γ‐Klotho so far. Recent research indicated that α‐Klotho, designated as Klotho, was involved in the progression of a variety of human cancers as a tumor suppressor, including hepatocellular carcinoma, colorectal cancer, renal cell carcinoma, pancreatic cancer, breast cancer, and lung cancer and so on.^16^ However, these conclusions are limited to experimental studies and a few clinical studies, mainly evaluating the tissue Klotho expression level. Studies on serum Klotho and human diseases are very limited, especially for cancer,^17^, ^18^, ^19^, ^20^, ^21^, ^22^ which report a lower Klotho expression in human hepatocellular carcinoma (20 cases vs 29 controls),^20^ but no significant difference in lung cancer (45 cases vs 43 controls).^22^

In addition to its anticancer effects, another important role of Klotho is acting as a hormone‐like factor.^13^ One population study found a positive correlation between plasma testosterone and secreted Klotho in healthy, sedentary middle‐aged men and women.^23^ Another in vitro experiment demonstrated upregulation of Klotho gene expression in 3T3‐L1 adipocytes during adipocytes differentiate.^24^ Testosterone had been reported to increase Klotho expression through an androgen receptor‐mediated pathway in animal studies.^25^ But the association between the Klotho and hormones in cancer has not been elucidated, and there is debate about the extent to which hormones mediate the value of Klotho's association with hormone‐related cancers.

Therefore, we will use serum Klotho levels from the US NHANES database to analyze its association with the risk of cancer, all‐cause mortality, and cancer‐specific mortality, as well as sex steroid hormones. This will be helpful to elucidate whether serum Klotho exerts a similar inhibitory effect on cancer as tissue, which may be through its interaction with sex hormones.

## 2 METHODS

### 2.1 Study population

The analysis data originated from the NHANES database, which uses interviews and physical examinations to assess the health and nutritional status of adults and children in the US. After 1999, the survey has been continuously conducted every two years as a cycle.^26^ The National Center for Health Statistics and the Research Ethics Review Committee of the Centers for Disease Control and Prevention approved the investigation plan. And written informed consent was provided by all participants or agents who were selected through a complex, multi‐stage probability sampling design.

**FIGURE 1:**
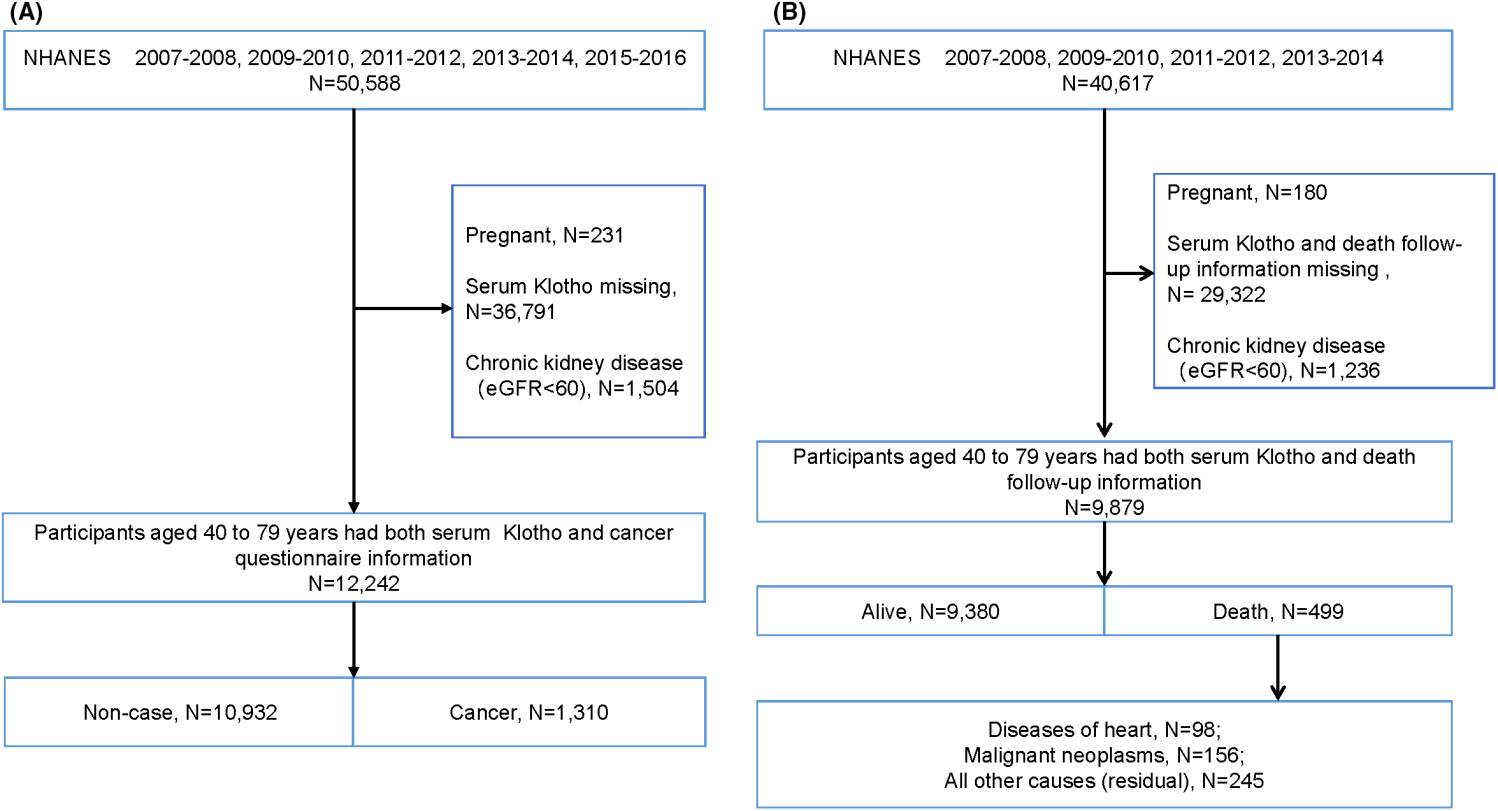
Flowchart displaying selection criteria of the study population. (A) Present population selection for analysis of the association between serum Klotho and cancer. (B) Population selection was presented to analyze the relationship between serum Klotho and all‐cause mortality and specific‐cause mortality. The 10th revised edition (ICD‐10) was based on the International Statistical Classification of Diseases and Related health problems, classified cause of death: diseases of heart (054–064), malignant neoplasms (019–043) or all other causes (Residual) (010)

In the analysis of the association between Klotho and the risk of cancer, we selected data of participants in NHANES 2007 to 2016 aged 40–79 years (only this age group was tested for serum Klotho level), and excluded participants pregnant, with chronic renal insufficiency (estimate glomerular filtration rate [eGFR] < 60 ml/min/1.73 m^2^), and without complete data of cancer questionnaire and serum Klotho level. Finally 12,242 participants were employed in the analysis, including 10,932 non‐case group and 1310 cancer patients (Figure 1A).

Regarding the relationship between Klotho and all‐cause mortality and cause‐ specific mortality, the participants were associated with mortality data from the survey date to December 31, 2015. Therefore, only the 2007–2014 population was selected, excluding pregnant, chronic renal insufficiency and missing data of serum Klotho and death follow‐up information. Among the 9879 participants eventually included in the study, according to the 10th edition of the World Health Organization's Internal Disease Classification (ICD‐10) coding standard, 499 participants died: 156 died of malignant tumors (C00‐C97) and 98 died of heart disease (I00‐I09, I11, I13, I20‐I51) during the 58‐month follow‐up period^27^ (Figure 1B).

### 2.2 Serum Klotho levels

Serum samples from participants were collected and stored at −80°C for later use. The detection was carried out by a commercially available ELISA kit produced by IBL International, Japan, with a sensitivity of 6 pg/ml. Repeated analyses of the same samples on four different days showed interanalysis coefficients of variation of 2.8% and 3.5% for the recombinant samples, compared with 3.8% and 3.4% for the human samples. In accordance with the manufacturer's protocol, the tester analyzed in duplicate, calculated their average values as final values, and performed laboratory standardization checks to ensure that all results were acceptable to the application prior to publishing the report. If there were values not within the 2 standard deviation (SD) range of the specified value for quality control samples, the entire analysis run would be rejected and the sample analysis would be repeated.^26^

### 2.3 Cancer information

The cancer status was assessed by two consecutive questions: first, if the participant answered “Yes” to the question of “Have a doctor or other health professional told you that you have cancer or any type of malignant tumor?”, he/she was classified as a cancer patient; next, the participant will be asked “What kind of cancer?”, and the type of cancer can be determined.

### 2.4 Sex steroid hormones

Testosterone and estradiol were measured by isotope dilution liquid chromatography tandem mass spectrometry (ID‐LC–MS/MS) in serum of subjects aged 6 years and older. The lower limits of testosterone and estradiol were 0.75 ng/ml and 2.994 pg/ml, respectively.

### 2.5 Covariates information

From demographics, examination, laboratory and questionnaire data, we selected the following potential confounding variables in our analysis: age (continuous), gender (male, female), race/ethnicity (non‐Hispanic white, non‐Hispanic black, Mexican American, others), education levels (under high school, high school, above high school), poverty income ratio (PIR) (0.00–1.30, 1.31–3.50, >3.51), body mass index (BMI, kg/m^2^) (normal<25, overweight 25–30, obese>30), smoking status (non‐smoking, passive smoking, active smoking), physical activity (low, moderate, high), alcohol intake (non‐drinking, low to moderate drinking, heavy drinking), diabetes (yes, no), cardiovascular diseases (yes, no), hypertension (yes, no), dyslipidemia (yes, no), self‐reported health (poor to fair, good, very good to excellent), total energy intake (low, adequate, high). The classification of some covariates was defined in the Table S1.

### 2.6 Statistical analysis

The NHANES database analysis tutorial recommends that all variables in the analysis be weighted with variables of interest that are appropriate to be collected on a minimum number of respondents (https://wwwn.cdc.gov/nchs/nhanes/tutorials/Module3.aspx). The sample weight from the laboratory mobile Examination Center (MEC) was used, as it contained a smaller sample size than the samples from the demographic and cancer questionnaires.

For the baseline data, continuous variables were expressed as median (Interquartile range, IQR) and analyzed by Wilcoxon test, and the remaining variables were expressed as column percentages and analyzed by weighted chi‐square analysis. We treated serum Klotho as a continuous variable and a categorical variable (tertiles) respectively. Weighted Logistic regression was used with Klotho as the exposure and cancer as the outcome, and all‐cause mortality was calculated by weighted Cox regression. The competitive risk model was calculated with cancer death as the outcome, non‐cancer death as the competitive event, and the others as deletion. And ordinal values were used in a separate Logistic model to test the cross‐tertiles trend test. Sensitivity analyses were further performed in the non‐cancer population to assess the robustness of the observed Klotho‐cancer specificity mortality relationship. The correlations between serum Klotho and testosterone and estradiol levels were analyzed by Spearman correlation and restricted cubic spline (RCS) respectively. All analyses were performed by *SAS 9.4*. A two‐sided *p* < 0.05 was statistically significant.

## 3 RESULTS

### 3.1 Baseline characteristics of study participants

The baseline characteristics of the study population are shown in Table 1. The median age of cancer patients was 64 years, much higher than the non‐case group (55 years). We found that participants of non‐Hispanic white, college or above, PIR > 3.51, low activity, active smoking, hypertension, cardiovascular disease, diabetes, dyslipidemia and low total energy intake were more likely to develop cancer. There was a significant difference in serum Klotho levels between cancer patients [median (IQR):95.53 (71.72, 125.45) ug/g creatinine] and non‐case group [median (IQR): 99.06 (76.73, 131.18) ug/g creatinine] (*p* = 0.0001) (Figure S1A), even the distribution of serum Klotho by tertiles did not differ between cancer and non‐case group (*p* = 0.4343) (Figure S1B).

**TABLE 1:** Baseline characteristics of study participants in NHANES 2007–2016

Variable	Total participant, *N*= 13,746	Non‐case, *N* = 12,144	Cancer, *N* = 1602	*p* value
Age, years, median (IQR)	12,242	55 (47, 63)	64 (56, 71)	<2.2e‐16
Age	12,242			<0.0001
40–59	7203	6774 (70.07)	429 (41.61)	
60–79	5039	4158 (29.93)	881 (58.38)	
Gender	12,242			0.1736
Male	5963	5348 (48.85)	615 (46.27)	
Female	6279	5584 (51.15)	695 (53.72)	
Ethnicity/race	12,242			<0.0001
Non‐Hispanic white	5241	4378 (70.88)	863 (87.03)	
Non‐Hispanic black	2206	2046 (9.07)	160(3.87)	
Mexican American	2046	1939 (7.62)	107 (2.39)	
Others	2749	2569 (12.42)	180 (6.70)	
Education	12,237			<0.0001
Less than High school or equivalent	3404	3141 (17.13)	263 (10.30)	
High school or equivalent	2693	2400 (22.32)	293 (19.38)	
College or above	6140	5386 (60.54)	754 (70.32)	
PIR	12,242			0.0024
0.00–1.30	4412	4003 (22.83)	409 (18.78)	
1.31–3.50	3993	3565 (30.54)	428 (29.37)	
>3.51	3837	3364 (46.63)	473 (51.84)	
BMI	12,242			0.4506
Normal < 25	3099	2752 (25.39)	347 (27.33)	
Overweight 25–30	4720	3843 (35.73)	427 (33.64)	
Obese>30	4873	4337 (38.87)	536 (39.03)	
Physical activity	11,958			0.0158
Low	8190	7283 (66.67)	907 (69.70)	
Moderate	2281	2046 (19.42)	235 (19.76)	
High	1487	1361 (13.91)	126 (10.54)	
Smoking status	12,242			0.0100
Non‐smoking	2920	2639 (23.83)	281 (21.01)	
Passive smoking	5793	5180 (43.27)	613 (41.32)	
Active smoking	3529	3113 (32.89)	416 (37.67)	
Alcohol drinking	11,526			0.0154
Non‐drinking	8711	7766 (71.47)	945 (70.26)	
Low to moderate drinking	973	841 (8.52)	132 (11.89)	
Heavy drinking	1842	1659 (20.01)	183 (17.85)	
Hypertension	12,242			<0.0001
No	6839	6237 (61.28)	602 (51.74)	
Yes	5403	4695 (38.72)	708 (48.25)	
Cardiovascular diseases	12,241			<0.0001
No	10,643	9596 (90.05)	1047 (83.130)	
Yes	1598	1335 (9.95)	263 (16.87)	
Diabetes	12,242			0.0077
No	9559	8579 (84.07)	980 (80.95)	
Yes	2683	2353 (15.92)	330 (19.05)	
Dyslipidemia	12,242			0.2768
No	5488	4883 (43.301)	605 (43.67)	
Yes	6754	6049 (56.69)	705 (56.33)	
Self‐reported health	11,355			0.1926
Poor to fair	3024	2683 (17.92)	341 (18.55)	
Good	4528	4023 (38.48)	505 (41.37)	
Very good to excellent	3803	3391 (43.60)	412 (40.08)	
Total energy intake	12,242			0.0064
Low	5739	5094 (40.66)	645 (44.96)	
Adequate	4384	3903 (39.22)	481 (39.65)	
High	2119	1935 (20.12)	184 (15.38)	

*Note*: The continuous variables were analyzed by Wilcoxon test, expressed by the median (IQR); the weighted chi‐square test was used to analyze the categorical variables, expressed by the column percentage. A level of two‐sided *p* < 0.05 was considered statistically significant.

Abbreviations: BMI, body mass index; IQR, Interquartile range; NHANES, National Health and Nutrition Examination Survey; PIR, poverty income ratio; SD, standard deviation.

### 3.2 Association of serum Klotho levels with the risk of cancers: NHANES 2007–2016

**FIGURE 2:**
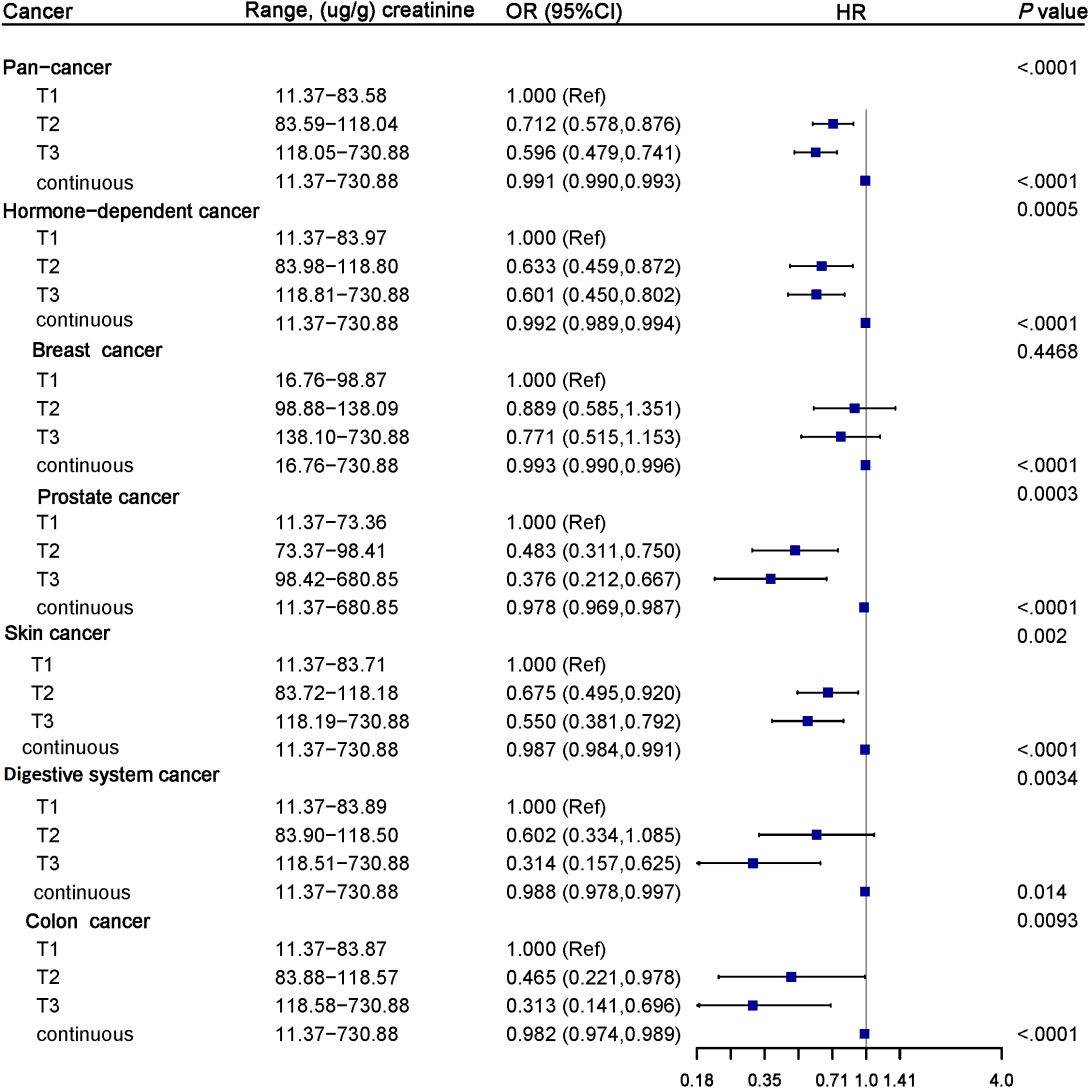
Forest plot for the association of serum Klotho with the risk of pan‐cancer and individual cancers from NHANES 2007–2016. Adjusted for age (continuous), gender, race/ethnicity, education levels, PIR, BMI, smoking status, physical activity, alcohol intake, diabetes, cardiovascular diseases, hypertension, dyslipidemia, self‐reported health, total energy intake. Abbreviations: CI, confidence interval; NHANES, National Health and Nutrition Examination Survey; OR, odds ratio

The association between serum Klotho levels and the risk of cancer was analyzed by weighted multifactor Logistic regression as a categorical variable and a continuous variable, respectively, and the results were shown in Figure 2. We found that Klotho, as a categorical variable, had a negative association with pan‐cancer (*p* < 0.0001), hormone‐related cancer (including prostate cancer and testicular cancer, breast cancer, cervical cancer, endometrial cancer, ovarian cancer and thyroid cancer) (*p* = 0.0005), prostate cancer (*p* = 0.0003), skin cancer (*p* = 0.002), digestive system cancer (including esophageal cancer, stomach cancer, pancreatic cancer, hepatocellular carcinoma, colon cancer and rectal cancer) (*p* = 0.0034), and colon cancer (*p* = 0.0093). Compared with serum Klotho in the first tertile, serum Klotho in the second tertile and third tertile were related to a decreased risk of these cancers, except for the breast cancer (*p* = 0.4468) and the second tertile of digestive cancer [OR (95%CI):0.602 (0.334,1.085)]. Moreover, we did a further trend test for serum Klotho and cancer, based on the OR value, and found that with the increase of serum Klotho level, its negative effect on these cancers was significantly enhanced [cancer (*p‐*_trend_): pan‐cancer (*p‐*_trend_ <0.0001), hormone‐related cancer (*p‐*_trend_ <0.0001), prostate cancer (*p‐*_trend_ <0.0001), skin cancer (*p‐*_trend_ <0.0001), digestive system cancer (*p‐*_trend_ <0.0001), colon cancer (*p‐*_trend_ = 0.0002)]. As a continuous variable, the effect of serum Klotho on all the cancers covered in this study showed similar results (all *p* < 0.02), with each unit increase in serum Klotho (1 ug/g creatinine) associated with a 0.9%–2.2% reduction in the risk of cancers.

In addition, we analyzed the quantitative response relationship between serum Klotho and the risk of cancer using RCS, and found no non‐linear relationship (all *p* > 0.05) (Figure S2).

### 3.3 Stratified analysis

We did a stratified analysis and found some potential interactions between serum Klotho level and age, sex, race/ethnicity and BMI in klotho‐cancer correlation (*p*‐interactions as <0.0001, 0.0006, <0.0001, <0.0001, respectively) (Table 2). In weighted multifactor Logistic regression, after adjusting for covariates, cancer was less likely to develop in populations with higher levels of Klotho at age 60–79 [T, OR (95CI): T2, 0.603 (0.425, 0.854); T3, 0.585 (0.415, 0.823)], in females [T, OR (95CI): T2, 0.718 (0.547,0.942); T3, 0.627 (0.485,0.811)] and in overweight people [T, OR (95CI): T2, 0.649 (0.485,0.867); T3, 0.564 (0.386,0.825)], but not in non‐Hispanic blacks (Table 2).

**TABLE 2:** Stratified analysis for the association between serum klotho and risk of cancer from NHANES 2007–2016

Klotho (ug/g creatinine)	Non‐case median (IQR)	Cancer median (IQR)	T1 (11.37–83.58)	T2 (83.59–118.04)		T3 (118.05–730.88)		*p‐interaction*
			OR (95% CI)	OR (95% CI)	*p‐*value	OR (95% CI)	*p*‐value	
Stratification factor								
Age								<0.0001
40–59	101.49 (78.72, 134.70)	107.12 (81.35, 134.08)	1.000	0.933 (0.644, 1.352)	0.7143	0.774 (0.550, 1.088)	0.1404	
60–79	95.97 (93.77, 125.87)	91.84 (68.52, 120.68)	1.000	0.603 (0.425, 0.854)	0.0045	0.585 (0.415, 0.823)	0.0021	
Gender								0.0006
Male	85.46 (67.40, 108.04)	79.41 (62.49, 103.41)	1.000	0.741 (0.568, 0.967)	0.0273	0.670 (0.485, 0.926)	0.0153	
Female	116.59 (90.16, 151.71)	112.22 (87.31, 142.31)	1.000	0.718 (0.547, 0.942)	0.0166	0.627 (0.485, 0.811)	0.0004	
Race/ethnicity								<0.0001
Non‐Hispanic white	93.85 (74.74, 120.92)	92.97 (70.70, 120.52)	1.000	0.762 (0.599, 0.969)	0.0268	0.655 (0.507, 0.846)	0.0012	
Non‐Hispanic black	96.16 (70.52, 96.16)	91.68 (67.33, 130.00)	1.000	0.640 (0.392, 1.046)	0.0751	0.657 (0.420, 1.028)	0.0661	
Mexican American	106.95 (81.15, 142.62)	107.50 (82.98, 147.14)	1.000	0.553 (0.330, 0.928)	0.0248	0.402 (0.210, 0.770)	0.0059	
Others	106.32 (81.69, 139.39)	102.72 (79.10, 138.84)	1.000	0.616 (0.373, 1.017)	0.0583	0.557 (0.325, 0.954)	0.0332	
BMI								<0.0001
Normal < 25	104.37 (80.37, 137.69)	97.23 (76.14, 129.03)	1.000	0.675 (0.453, 1.004)	0.0524	0.580 (0.408, 0.824)	0.0024	
Overweight 25–30	95.78 (74.40, 125.34)	92.82 (67.23, 123.66)	1.000	0.649 (0.485, 0.867)	0.0035	0.564 (0.386,0.825)	0.0031	
Obese > 30	99.33 (76.49, 132.14)	95.90 (71.98, 125.53)	1.000	0.796 (0.588, 1.078)	0.1403	0.615 (0.432, 0.878)	0.0073	

*Note*: Adjusted for age (continuous) (not for age stratification analysis), gender (not for gender stratification analysis), race/ethnicity (not for ethnic stratification analysis), education levels, PIR, BMI (not for BMI stratification analysis), smoking status, physical activity, alcohol intake, diabetes, cardiovascular diseases, hypertension, cancer, dyslipidemia, self‐reported health, total energy intake.

Abbreviations: CI, confidence interval; IQR, interquartile range; OR, odds ratio.

### 3.4 Association of serum Klotho levels with mortality: NHANES 2007–2014

**FIGURE 3:**
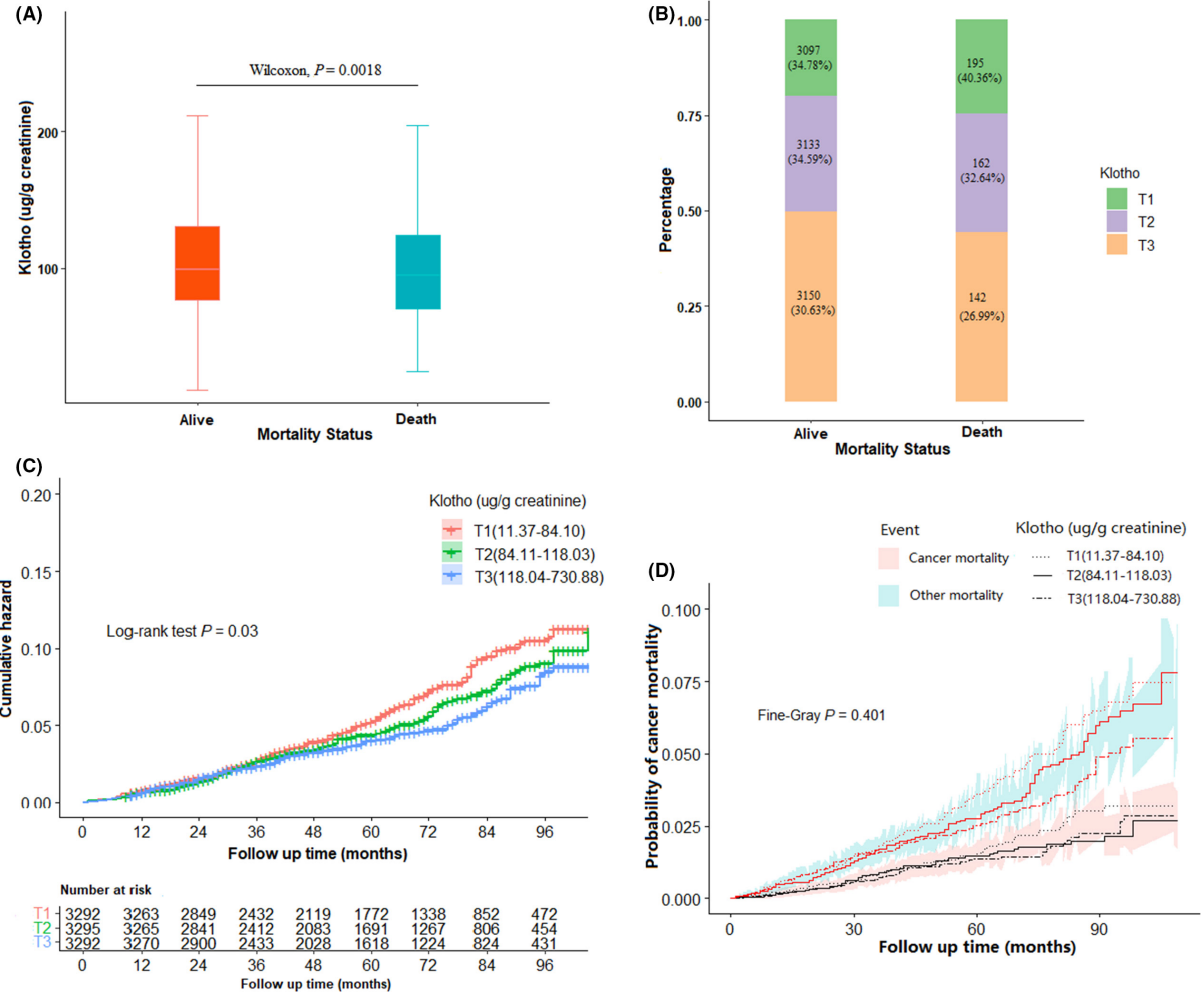
The association of serum Klotho with mortality. (A) Klotho level by mortality status. (B) Distribution of Klotho by mortality status. (C) Cumulative risk of death by Klotho level. (D) Probability of cancer mortality by Klotho level

First, we analyzed the distribution of serum Klotho between mortality state using Wilcoxon test and found higher Klotho levels in the living than in the dead [Figure 3A (*p* = 0.0018) and 3B (*p* = 0.1342)]. Then our analysis showed that participants with serum Klotho in the third tertile had a lower risk of death compared to that in the first tertile (log‐rank test, *p*= 0.03, Figure 3C). Similarly, we did not find that serum Klotho contributed to the cancer‐specific mortality by Fine‐Gray test used to calculate the probability of cancer‐specific mortality (*p*= 0.401, Figure 3D). Finally, after adjusting covariates, there were no association between categorical serum Klotho levels with cancer‐specific mortality (*p*‐trend = 0.8311), heart disease mortality (*p*‐trend = 0.8754) and all‐cause mortality (*p*‐trend = 0.9461). Similar results were observed when Klotho was used as a continuous variable (Table S2). Further, we did a sensitivity analysis for the association of Klotho and mortality in non‐case participants, and similar results were found (Table S3).

### 3.5 Correlation between serum Klotho and sex steroid hormones: NHANES 2013–2016

**FIGURE 4:**
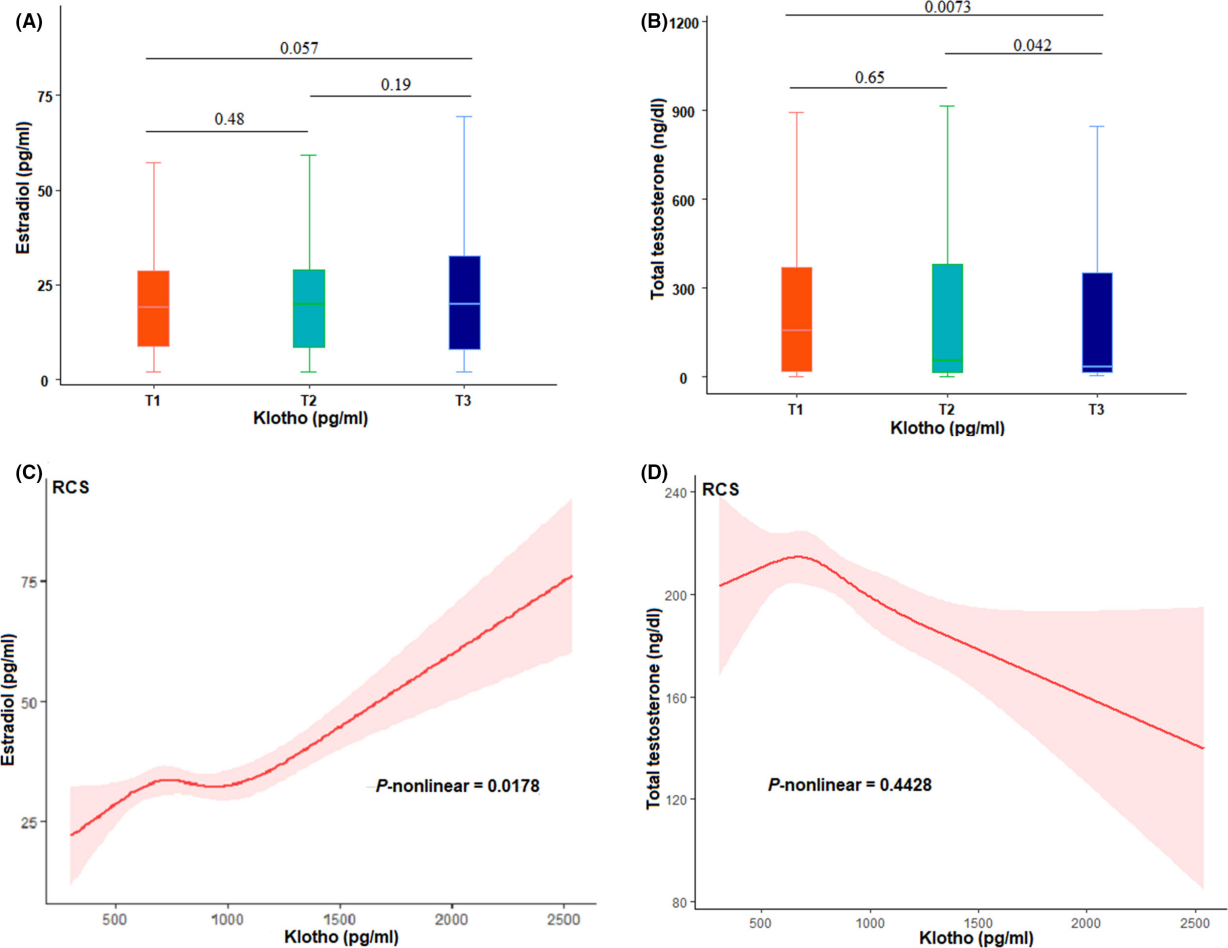
Correlation between serum Klotho and sex steroid hormones: NHANES 2013–2016. (A and B) distribution of estradiol and testosterone by Klotho level. (C and D) restricted cubic spline analysis of the nonlinear relationship between continuous Klotho in estradiol and testosterone

Since Klotho itself is a circulating hormone and the higher level of serum Klotho had a strong negative effect on hormone‐related cancers, we speculated that serum Klotho might play this role by influencing the level of sexual steroid hormones. Therefore, we analyzed the correlation between serum Klotho and sexual steroid hormones using data from NHANES 2013–2016. First, we analyzed the distribution of estradiol and testosterone level by serum Klotho respectively, and found no difference between estradiol and Klotho level (Figure 4A). Whereas, testosterone level was lower in the third tertile level of serum Klotho when compared to the first and second tertile level (*p* = 0.0037 and *p* = 0.042, respectively, Figure 4B). The Spearman correlation analysis was performed and a marginal positive correlation was found between serum Klotho and estradiol (β = 0.03, *p* = 0.0541), but a weak negative correlation was found between serum Klotho and testosterone (β = −0.04, *p* = 0.0024). A nonlinear positive correlation between serum Klotho and estradiol was found by using RCS analysis (*p*‐nonlinear = 0.0178, Figure 4C), which was not observed for testosterone (*p*‐nonlinear = 0.4428, Figure 4D). We subsequently explored the correlation between Klotho and testosterone by linear regression. In a single‐factor linear regression, Klotho and testosterone were found to be negatively correlated, but in multi‐factor regression, a positive correlation was observed between them, possibly caused by gender. Further, gender‐stratified analysis confirmed a positive association between testosterone and Klotho only in male participants (Table S4).

## 4 DISCUSSION

In this large‐scale population‐based database of NHANES, we explored comprehensively the effect of serum Klotho on cancer, and found regarding pan‐cancer, there was no doubt that serum Klotho exerted a tumor suppressor effect, and a higher level of Klotho concentration had a stronger effect. According to the results of stratified analysis, the negative association of serum Klotho with pan‐cancer was significantly different by age, gender, BMI and racial/ethnic stratification, with people aged 60 to 79 years, females, overweight and non‐Hispanic whites or Mexican Americans less likely to develop cancer.

For hormone‐related cancer, prostate cancer, breast cancer, digestive and colon cancer, similar negative effect was observed with increased serum Klotho levels, although the tertiles of serum Klotho was not found to be associated with breast cancer risk, but this does not affect that there was consistent with previous histological evidence that Klotho is a tumor inhibitor.^28^, ^29^, ^30^, ^31^, ^32^ A meta‐analysis showed that the F allele of the F352 V polymorphism in Klotho gene protects against breast and ovarian cancer susceptibility and is associated with an overall risk of cancer in BRCA1 mutation carriers.^33^ F352 V polymorphic mutation has been reported to alter protein function by affecting Klotho transport and catalytic activity in HeLa cells.^34^ There are few studies on the relationship between Klotho and prostate cancer. One study has shown that the C1548T polymorphism of Klotho (KL) gene was associated with prostate cancer.^35^ Also, two studies have showed that Klotho variants (rs1207568, rs564481 and G‐395A) are associated with colorectal cancer risk.^36^, ^37^ Further, mutations in G‐395A AA and GA genotypes in the promoter region of Klotho gene down‐regulate tissue Klotho expression in colorectal cancer.^38^ All of the above demonstrate the tumor suppressor effect of Klotho, and our analysis further supplemented the evidence. In addition, since no research on skin cancer and Klotho has been found yet, we suspect that this may be the first to find an association between serum Klotho and skin cancer.

Regarding the association between serum Klotho and all‐cause mortality and cancer mortality, we found no association Klotho in multivariate analysis. Our results were inconsistent with those of Jacob K. Kresovich et al., who found an inverse relationship between serum Klotho concentration and mortality using the same database.^39^ The main reason was that we overcame some defects: Firstly, since Klotho was mainly produced in the kidney, its function would directly interfere with the content of Klotho, which may affect the association between Klotho and cancer to some extent. Therefore, we excluded participants with chronic kidney disease and corrected Klotho with serum creatinine. Secondly, when analyzing individual causes of death, we chose the competitive risk model compared with Cox regression model, and the results would be more reliable. Finally, studies have found that patients with cardiovascular disease, hypertension, cancer and dyslipidemia have a higher risk of death, which we consider as a covariable to be included in the analysis. Overall, our results were more robust and provided further evidence for the association between Klotho and mortality.

Considering the strong association between Klotho and hormone‐related tumors, we further analyzed the correlation between Klotho and estradiol and testosterone, and found non‐linear correlation between Klotho and estradiol. Although there was a plateau in the middle, the overall trend was that with the increase of Klotho level, the estradiol level also increased. Further, gender‐stratified analysis confirmed a positive association between testosterone and Klotho only in male participants. Previous studies have shown that 17β ‐estradiol prevents Klotho deficiency from inducing heart failure by eliminating upregulation of Na‐Pi cotransporter expression in the kidneys of female mice.^40^ Manuel Dote Montero et al. found a positive correlation between Klotho and testosterone in women in a randomized controlled trial.^23^ Another mice study showed that testosterone up‐regulated anti‐aging Klotho expression in the kidney by recruiting androgen receptors to the androgen response element of the Klotho promoter.^25^ At present, the research on this aspect was very limited. The existing research had the limitation of few samples, and more subsequent studies are needed to support this view.

This was the first time that we analyzed the association between serum Klotho and cancer in a large‐scale population study of a nationally representative sample. Our findings showed that serum Klotho presented a negative association with cancer. This adds to the growing body of evidence linking Klotho as a new biomarker to cancer and may open new possibilities for cancer diagnosis and treatment. After all, serum is easier to obtain and has less harm to the human body. At the same time, our analysis had certain limitations. First, our analysis was a cross‐sectional analysis, which was not enough to provide stronger evidence. Secondly, cancer information was obtained from questionnaire surveys, and we cannot guarantee that the data was completely credible. Finally, the 2007–2016 NHANES lacks cancer markers and related clinico‐pathological information, as well as the lack of relevant genotypic information, which hampered further analysis.

## 5 CONCLUSIONS

In summary, our analysis showed that higher levels of serum Klotho were negatively associated with risk of pan‐cancer, but not with cancer‐specific mortality. More and more comprehensive information was needed to analyze the association between serum Klotho and cancer.

### AUTHOR CONTRIBUTION

Yating Qiao, Fubin Liu, Yu Peng and Peng Wang: Methodology, formal analysis, data curation, visualization. Bing Ma, Limin Li, Changyu Si and Xixuan Wang: writing—review & editing. Ming Zhang and Fangfang Song: Conceptualization, methodology, formal analysis, resources, writing—original draft, writing—review & editing. Yating Qiao analyzed the data and wrote the manuscript. All authors interpreted the results and contributed to critical review of the manuscript. All authors gave final approval of the version to be published; agreed on the journal to which the article has been submitted; and agree to be accountable for all aspects of the work.

## CONFLICT OF INTEREST

All authors have made no disclosure.

## ETHICS STATEMENT

The manuscript of the data from the NHANES database (https://www.cdc.gov/nchs/nhanes/about_nhanes.htm), and the National Center for Health Statistics and the Research Ethics Review Committee of the Centers for Disease Control and Prevention approved the investigation plan.

## Supporting information


Table S1
Table S2
Table S3
Table S4
Figure S1
Figure S2

## Data Availability

I confirm that I have included a citation for available data in my references section, unless my article type is exempt.
