# Supplementary material for: Association of serum Klotho levels with cancer and cancer mortality: Evidence from National Health and Nutrition Examination Survey
Source: Cancer Med. 2022 Jul 16;12(2):1922–34. doi: 10.1002/cam4.5027 (PMC9883546; doi:10.1002/cam4.5027)
Supplement: Supplementary file 1 — Table S1 Table S2 Table S3 Table S4 Figure S1 Figure S2 [file CAM4-12-1922-s001.docx]

**Supplementary Materials**

**Supplementary Table 1.** **Defined variables.**

**Supplementary Table 2. The distribution and association between serum klotho and all-cause mortality and cancer-specific mortality from NHANES 2007-2014.**

**Supplementary Table 3. Sensitivity analysis was performed in non-case participants from NHANES 2007-2016.**

**Supplementary Table 4. The association between serum klotho, testosterone and hormone-related cancers from NHANES 2013-2016.**

**Supplementary Figure 1. Distribution of serum Klotho in cancer and non-case group.**

**Supplementary Figure 2. Restricted cubic splines for dose-response relationships between serum Klotho and pan-cancer and individual cancers.**

**Supplementary Table 1. Defined variables.**

| ***Variable*** | **Classification** | **Definition** |
| --- | --- | --- |
| ***Physical activity*** | Low | No activity is reported or some activity is reported but not enough to Moderate High and moderate categories. |
|  | Mod-rate | Either of the following three criteria:  (a) 3 or more days of vigorous-intensity of at least 20 minutes per day;  or(b) 5 or more days of moderate-intensity and/or walking of at least 30 minutes per day or(c) 5 or more days of any combination of walking, moderate-or vigorous-intensity activities accumulating at least 600 MET-minutes/week. |
|  | High | Participants have at least 3 days of vigorous-intensity activity and accumulate at least 1500 MET minutes per week or 7 or more days of walking, moderate or vigorous-intensity activities in any combination, and accumulate at least 3000 MET minutes per week. |
| ***Smoking*** | Non- smoking | Cotinine<0.011 ng/ml; |
|  | Passive smoking | 0.011 ng/ml<= Cotinine<10 ng/ml; |
|  | Active smoking | Cotinine>=10 ng/ml. |
| ***Alcohol intake*** | Non- drinking | Non- drinking was categorized as none (0 g/d); |
|  | Low to Moderate drinking | Low to Moderate drinker (0.1 to 27.9 g/d for men and 0.1 to 13.9 g/d for women); |
|  | Heavy drinking | Heavy drinking (≥28 g/d for men and ≥14 g/d for women). |
| ***Hypertension*** | No | - |
|  | Yes | The average of three consecutive measurements in SBP and DBP is calculated. Hypertension is defined by mean SBP ≥ 140 mmHg or mean DBP ≥ 90 mmHg or antihypertensive medication user or physician diagnosis. |
| ***Cardiovascular diseases*** | No | - |
|  | Yes | A doctor’s diagnosis of congestive heart failure or coronary heart disease or heart attack or myocardial infarction or angina or stroke is defined as cardiovascular disease. |
| ***Diabetes*** | No | - |
|  | Yes | Diabetes through the participant’s fasting blood glucose ≥126 mg/dL or hemoglobin A1c ≥ 6.5% or random blood glucose ≥200 mg/dL, or being told by a doctor or health professional that you have diabetes or sugar diabetes or are taking insulin or hypoglycemic drugs. |
| ***Dyslipidemia*** | No | - |
|  | Yes | Participants' TG (≥150 mg/dL), TC (≥200 mg/dL), LDL-C (≥130 mg/dL) non-HDL-C (≥160 mg/dL), and Apo B (≥130 mg/dL) or taking drugs to treat dyslipidemia. |
| ***Total energy intake*** | Low | Males <2000 kcal/day, females <1600 kcal/day |
|  | Adequate | Males 2000–3000 kcal/day, females 1600–2400 kcal/day |
|  | High | Males >3000 kcal/day, females >2400 kcal/day |

Abbreviation: METs, metabolic equivalents.

**Supplementary Table 2. The association between serum klotho and all-cause mortality and cause-specific mortality from NHANES 2007-2014.**

|  | **Alive (N=9,380)** | | **Death**  **(N=499)** | | **P-value** | **All-cause mortality** | | **Cancer mortality** | |
| --- | --- | --- | --- | --- | --- | --- | --- | --- | --- |
|  |  |  |  |  |  | **HR (95% CI)** | ***P*-value** | **HR (95% CI)** | ***P*-value** |
| **Klotho, ug/g creatinine** |  | | |  | 0.1349* |  |  |  |  |
| T1(<84.10) | 3099 (34.79%) | | | 195 (40.36%) |  | 1.000 |  | 1.000 |  |
| T2(84.11–118.03) | 3131 (34.58%) | | | 162 (32.64%) |  | 0.975 (0.712,1.336) | 0.8732 | 1.041 (0.677,1.600) | 0.8555 |
| T3(>730.88) | 3150 (30.63%) | | | 142 (26.99%) |  | 1.017 (0.702,1.435) | 0.9244 | 0.899 (0.590,1.369) | 0.6191 |
| P-trend | |  | |  |  |  | 0.9461 |  | 0.8311 |
| **Klotho, ug/g creatinine** | 96.85 (76.56,127.12) | | | 96.72 (76.42,126.95) | 0.0018^#^ | 1.001 (0.998,1.004) | 0.6035 | 1.001 (0.997,1.004) | 0.7472 |

The continuous variables were analyzed by Wilcoxon test, expressed by the Median (Interquartile range, IQR); the weighted chi-square test was used to analyze the categorical variables, expressed by the column percentage.

Adjusted for age (continuous), gender, race/ethnicity, education levels, PIR, BMI, smoking status, physical activity, alcohol intake, diabetes, cardiovascular diseases, hypertension, cancer, dyslipidemia, self-reported health, total energy intake.

The Cox proportional hazard model is used for the analysis of all-cause mortality, and the competitive risk model is used for the analysis of cancer and heart disease mortality.

^#^: *P* value from the Wilcoxon test. *: *P* value from the weighted chi-square test.

**Supplementary Table 3. Sensitivity analysis was performed in non-case participants from NHANES 2007-2016.**

|  | **Alive (N=8,416)** | **Death (N=392)** | **P-value** | **All-cause mortality** | **Cancer mortality** | |  |
| --- | --- | --- | --- | --- | --- | --- | --- |
|  |  |  |  | **HR (95% CI)** | ***P*-value** | **HR (95% CI)** | ***P*-value** |
| **Klotho, ug/g creatinine** |  |  | 0.3703* |  |  |  |  |
| T1(<84.30) | 2788 (35.04%) | 147 (39.56%) |  | 1.000 |  | 1.000 |  |
| T2(84.31-118.48) | 2804 (34.45%) | 133 (33.14%) |  | 1.046 (0.737,1.486) | 0.7976 | 1.022 (0.618,1.690) | 0.9318 |
| T3(>118.49) | 2824 (30.51%) | 112 (27.30%) |  | 1.115 (0.748,1.662) | 0.5889 | 1.148 (0.680,1.939) | 0.6061 |
| ***P*-trend** |  |  |  |  | 0.5911 |  | 0.6117 |
| **Klotho, ug/g creatinine** | 99.29 (77.43,131.25) | 99.15 (77.27,131.01) | 0.0021^#^ | 1.002 (0.999,1.005) | 0.2212 | 1.001 (0.998,1.005) | 0.4878 |

The continuous variables were analyzed by Wilcoxon test, expressed by the Median (Interquartile range, IQR); the weighted chi-square test was used to analyze the categorical variables, expressed by the column percentage.

Adjusted for age (continuous), gender, race/ethnicity, education levels, PIR, BMI, smoking status, physical activity, alcohol intake, diabetes, hypertension, dyslipidemia, cardiovascular diseases, self-reported health, total energy intake.

The Cox proportional hazard model is used for the analysis of all-cause mortality, and the competitive risk model is used for the analysis of cancer and heart disease mortality.

^#^: *P* value from the Wilcoxon test. *: *P* value from the weighted chi-square test.

**Supplementary Table 4. The association between serum klotho, testosterone and hormone-related cancers from NHANES 2013-2016.**

| **Klotho** | **Testosterone** | | | |
| --- | --- | --- | --- | --- |
|  | **Univariate linear regression** | | **Multifactor linear regression*** | |
|  | **β (95%CI)** | ***p*** | **β (95%CI)** | ***p*** |
| **Linear regression** | | | | |
|  | -1.290 (-1.541, -1.039) | <.0001 | 0.154 (0.017,0.292) | 0.0289 |
| **Stratified analysis (gender)** | | | | |
| **Male** | 0.474 (0.080,0.89) | 0.02 | 0.513 (0.113,0.9140 | 0.0137 |
| **Female** | 0.007 (-0.020,0.034) | 0.5989 | 0.011 (-0.028,0.051) | 0.5674 |

Adjusted for age (continuous), gender, race/ethnicity, education levels, PIR, BMI, smoking status, physical activity, alcohol intake.


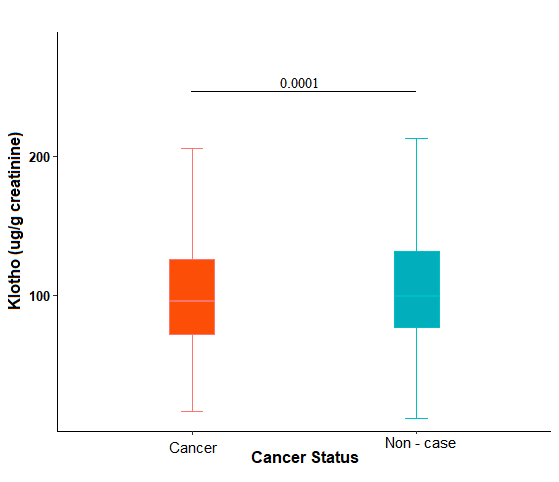

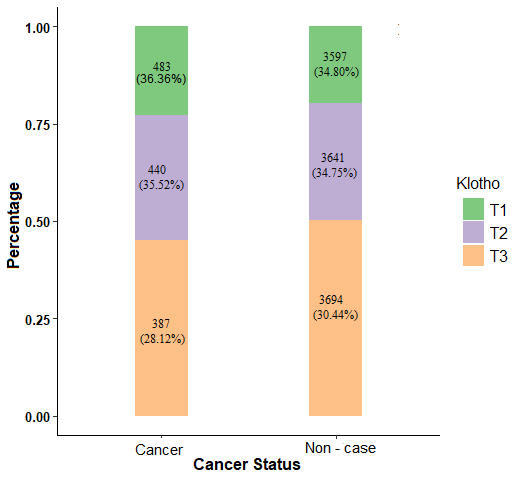


B

A

**Supplementary Figure 1. Distribution of serum Klotho level (A) as continuous variable and (B) as categorical variable in cancer and non-case group.**

**
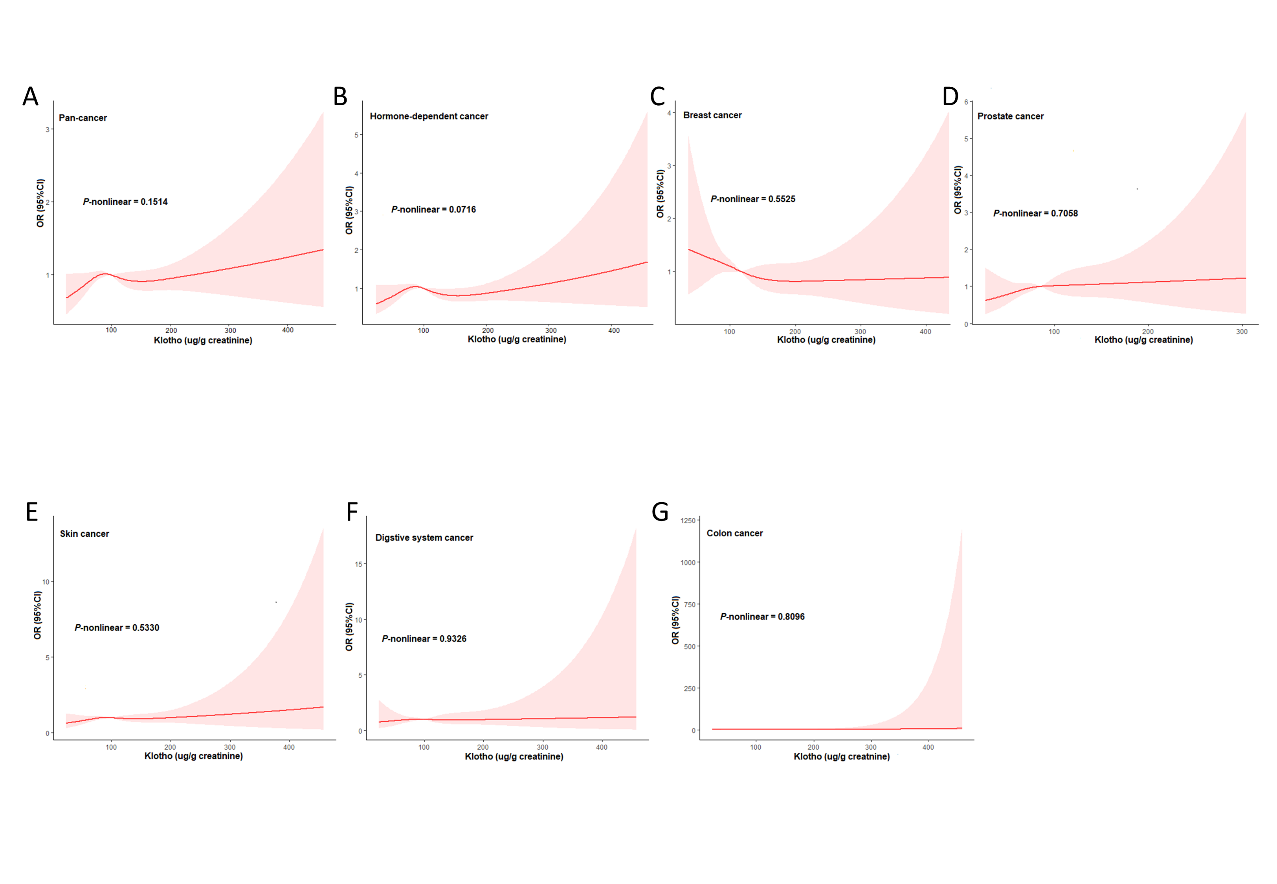
**

**Supplementary Figure 2. Restricted cubic splines for dose-response relationships between serum Klotho and pan-cancer and individual cancers.**

Adjusted for age, gender, race/ethnicity, education levels, PIR, BMI, smoking status, physical activity, alcohol intake, diabetes, cardiovascular diseases, hypertension, dyslipidemia, self-reported health, total energy intake.

Abbreviations: OR, odds ratio; CI, confidence interval; NHANES, National Health and Nutrition Examination Survey.
